# CAMBRA Protocol Efficacy: A Systematic Review and Critical Appraisal

**DOI:** 10.3390/dj10060097

**Published:** 2022-06-01

**Authors:** Ana Coelho, Inês Amaro, Tainá Iunes, Anabela Paula, Carlos Miguel Marto, José Saraiva, Manuel Marques Ferreira, Eunice Carrilho

**Affiliations:** 1 Institute of Integrated Clinical Practice, Faculty of Medicine, University of Coimbra, 3000-075 Coimbra, Portugal; ines.amaros@hotmail.com (I.A.); taiunes@gmail.com (T.I.); anabelabppaula@sapo.pt (A.P.); ze-93@hotmail.com (J.S.); eunicecarrilho@gmail.com (E.C.); 2 Area of Environment Genetics and Oncobiology (CIMAGO), Faculty of Medicine, Coimbra Institute for Clinical and Biomedical Research (iCBR), University of Coimbra, 3000-548 Coimbra, Portugal; cmiguel.marto@uc.pt (C.M.M.); m.mferreira@netcabo.pt (M.M.F.); 3 Clinical Academic Center of Coimbra (CACC), 3004-561 Coimbra, Portugal; 4 Institute of Biophysics, Faculty of Medicine, University of Coimbra, 3004-548 Coimbra, Portugal; 5 Institute of Experimental Pathology, Faculty of Medicine, University of Coimbra, 3004-548 Coimbra, Portugal; 6 Institute of Endodontics, Faculty of Medicine, University of Coimbra, 3000-075 Coimbra, Portugal

**Keywords:** dental caries, prevention, oral health, cariogenic bacteria

## Abstract

The Caries Management by Risk Assessment (CAMBRA) protocol consists of analyzing the patient’s profile by assessing the risk and protective factors and assigning a risk level to the patient to allow an individualized treatment plan, which combines restorative treatments with a preventive chemical therapy. This systematic review and critical appraisal aim to evaluate the effectiveness of the CAMBRA protocol in reducing the incidence of caries lesions and oral bacterial load. An electronic search was carried out in the Cochrane Library, PubMed, Web of Science, Scopus, and Embase databases. Clinical studies evaluating the incidence of dental caries lesions and/or the reduction of cariogenic bacterial load (*Streptococcus mutans* and/or *Lactobacilli* spp.), with at least an intervention group that follows the CAMBRA protocol and a control group published up until January 2022, were included. The methodological quality assessment of the included clinical studies was assessed through the Revised Cochrane risk-of-bias tool for randomized trials (RoB 2). The quality of the case-control study was evaluated according to the Methodological Index for Non-Randomized Studies (ROBINS-I). There is currently no scientific evidence to recommend the integration of the CAMBRA protocol into clinical practice. The results reported by the studies included in the systematic review do not suggest a decrease in the incidence of dental caries lesions and/or bacterial load. There is a clear need for the development of new clinical studies with an adequate methodology and follow-up, both for the CAMBRA protocol and for its components individually.

## 1. Introduction

Dental caries is one of the most common diseases worldwide and the main cause of tooth loss, affecting all ages. It is a multifactorial pathology that occurs due to an imbalance among the host, the environment, and the oral microbiome, resulting in the demineralization of the tooth structure [1,2,3,4,5,6].

Although signs of dental demineralization are identified in dental tissues, dental caries has its origin in the dental biofilm, being affected by several factors, such as salivary flow, oral hygiene habits, exposure to fluoride, or diet. The microbiological composition of dental biofilm is diverse and includes Gram-positive and Gram-negative bacteria and mostly anaerobic or facultative anaerobes. Of greater relevance for the development of dental caries lesions are *Streptococcus mutans* and *Lactobacillus* spp. [2,3].

Despite the significant prevalence of caries found in most populations, it has been decreasing in industrialized countries over the last few decades. Several studies find a higher prevalence of dental caries associated with populations of lower socioeconomic levels, which may be related to a lower access to information and/or education, worse dietary habits, and a lower access to prevention and treatment [1,4].

Restorative procedures of the dental structures affected by dental caries were for a long time seen as the only valid treatments of the pathology. However, restorative treatments per se are not capable of modifying the importance of other risk factors, a fact which makes the patient susceptible to multiple retreatments, secondary caries, and the appearance of new lesions [7,8].

Thus, the management of dental caries is currently planned according to a global concept, focusing not only on diagnosis, but also on identifying risk factors and developing an individualized treatment plan [9,10].

Thus, and in order to change the way in which the treatment of dental caries is conceptualized, the Caries Management by Risk Assessment (CAMBRA) [11] was created. The first protocol proposal, to be applied to both adults and children from 6 years of age, was published in 2007 [12,13]. In the same year, a protocol aimed at children under 6 was developed [14,15], and it was later updated in 2010. To date, the last protocol updates were added in 2019 [16] and 2021 [17].

The CAMBRA protocol consists of analyzing the patient’s profile by assessing risk factors (such as acidogenic bacteria, frequent snacking on fermentable carbohydrates, and low salivary flow rate) and protective factors (such as use of fluoride and antibacterial therapies). Then, a risk level is assigned to the patient (low, moderate, high, or extreme) in order to allow an individualized treatment plan, which combines restorative treatments with a preventive chemical therapy. The therapy may include fluoride (toothpaste, varnish, and/or mouthwash), a chlorhexidine mouthwash, sealants, and/or salivary enhancers. The frequency and application protocols of such therapies will depend on the patient’s age and caries risk assessment. The protocol provides all the necessary tools for its application, including a questionnaire to identify risk factors, risk indicators, and protective factors [18].

This systematic review aims to evaluate the effectiveness of the CAMBRA protocol in reducing the incidence of caries lesions and oral bacterial load.

## 2. Materials and Methods

The PICO methodology was used (Population, Intervention, Control, Outcome) [19]. Please see Table 1 below. 

This systematic review was registered in the International Prospective Register of Systematic Reviews (PROSPERO) platform (temporary ID: 316032) and designed according to the PRISMA guidelines (Preferred Reporting Items for Systematic Reviews and Meta-Analysis) [20].

### 2.1. Search Strategy

An electronic search was carried out in the Cochrane Library (www.cochranelibrary.com, accessed on 19 January 2022), PubMed (www.ncbi.nlm.nih.gov/pubmed, accessed on 19 January 2022), Web of Science (www.webofscience.com, accessed on 19 January 2022), Scopus (www.scopus.com, accessed on 19 January 2022), and Embase (www.embase.com, last accessed on 19 January 2022) databases for articles published until 19 January 2022, with no restrictions on the region or year of publication. The search keys used in the different databases are presented in Table 2.

### 2.2. Inclusion and Exclusion Criteria

Clinical studies evaluating the efficacy of the CAMBRA protocol were included. Inclusion and exclusion criteria are presented in Table 3.

### 2.3. Study Selection

The eligibility of the initially selected articles was evaluated by reading titles and abstracts. The full text of all potentially relevant articles was then checked for eligibility.

This process was carried out by two independent reviewers. Any disagreement was discussed and, if necessary, the opinion of a third reviewer was provided.

### 2.4. Data Extraction

For data extraction purposes, all of the selected articles were read independently by two reviewers. A Microsoft^®^ Excel (Microsoft, Washington, WA, USA) table was created in order to include relevant information: name of the authors, year of publication, type of study, groups (n), intervention, follow-up, dental caries incidence, and bacterial load.

### 2.5. Quality Assessment

The methodological quality assessment of the included clinical studies was assessed by two independent reviewers. Both randomized controlled trials were evaluated through the Revised Cochrane risk-of-bias tool for randomized trials (RoB 2) [21]. Five domains were evaluated: (D1) risk of bias arising from the randomization process; (D2) risk of bias due to deviations from the intended interventions; (D3) risk of bias due to missing outcome data; (D4) risk of bias in measurement of the outcome; and (D5) risk of bias in selection of the reported results. From this evaluation, each study may vary its overall classification regarding bias risk as “low”, “high”, or “some concerns”. The quality of the case-control study was evaluated according to the Methodological Index for Non-Randomized Studies (ROBINS-I) [22]. Seven domains were evaluated: (D1) bias due to confounding; (D2) bias due to selection of participants; (D3) bias in classification of interventions; (D4) bias due to deviations from intended interventions; (D5) bias due to missing data; (D6) bias in measurement of outcomes; and (D7): bias in selection of the reported result. From this evaluation, the observational study may vary its overall classification regarding bias risk as ”low”, “moderate”, “serious”, or “critical”.

## 3. Results

**Figure 1:**
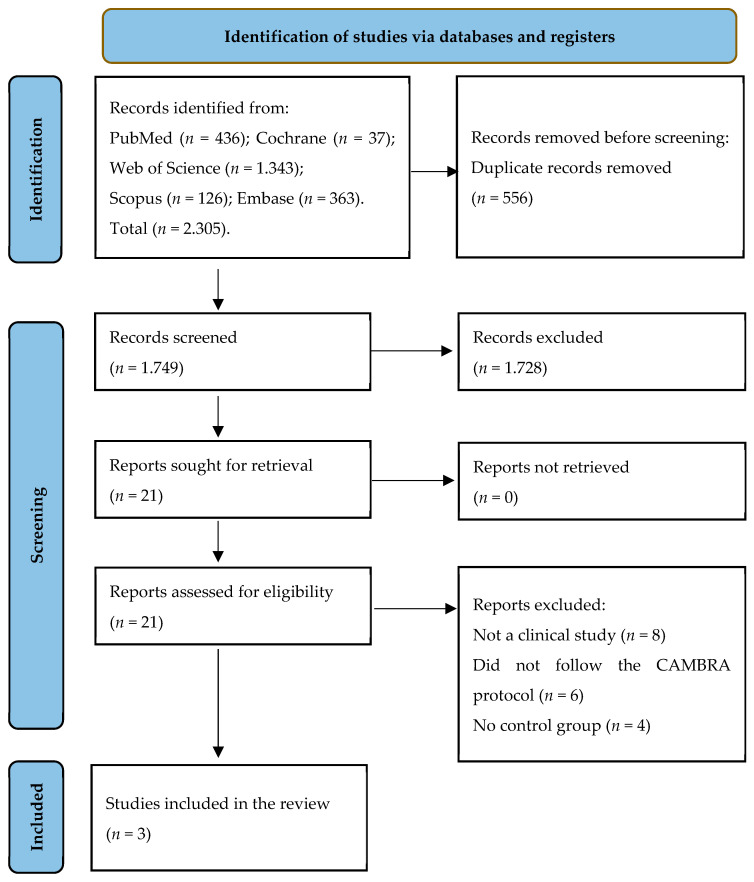
PRISMA flow diagram of screening and selection processes.

Initial screening of electronic databases yielded a total of 2305 articles. After elimination of duplicate studies, a total of 1749 titles and abstracts were assessed. A total of 21 potentially relevant articles were selected after an evaluation of titles and abstracts. The full text of these articles was thoroughly evaluated, and of these, three articles (two randomized controlled trials and one case-control study) met the inclusion criteria and were included in this systematic review. The flowchart of the data selection process is presented in Figure 1.

The included articles were published in 2015 [7], 2018 [23], and 2021 [24]. No authors used the 2019/2021 update of the CAMBRA protocol [16,17].

The samples of the included studies consisted only of adults, with a mean age between 36 [23] and 69 years old [24]. The sample sizes varied between 109 [7] and 460 [23], and the follow-up periods varied between 12 [24] and 24 months [7,23].

Of the three included studies, all authors reported results regarding the incidence of caries lesions, but only one [7] reported results regarding bacterial load.

Rechmann et al. [23] were the only authors to specify the protocol used in each group (control and intervention). In the control group, placebos were used, and for both groups the reported protocol varied according to the previously assessed caries risk.

Cheng et al. [7] were the only authors to report results with statistically significant differences. The intervention group had a lower caries incidence than the control group at 24 months and a lower salivary *Streptococcus mutans* load at 12 months.

The results are presented in Table 4.

**Figure 2:**
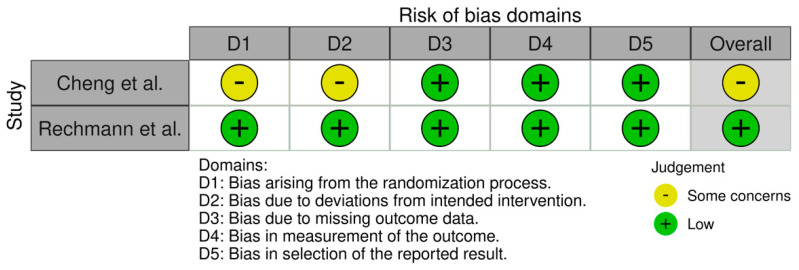
Methodological quality assessment of the included randomized controlled trials [7,23] using the Revised Cochrane risk-of-bias tool for randomized trials (RoB 2).

**Figure 3:**
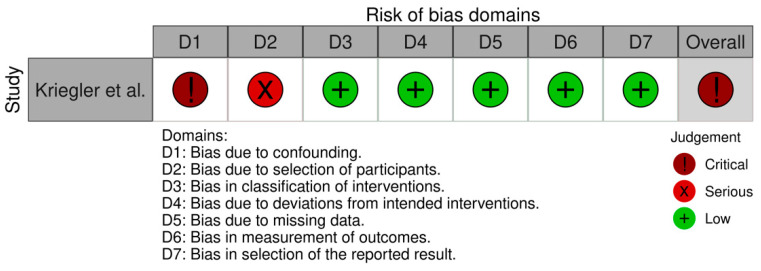
Methodological quality assessment of the included case-control study [24] using the Methodological Index for Non-Randomized Studies (ROBINS-I).

The results of the studies’ quality assessment are presented in Figure 2 and Figure 3.

One of the randomized controlled trials [7] presented an overall classification of “some concerns”, while the other one [23] presented an overall “low” risk of bias. As for the observational study [24], it presented an overall classification of “critical”. In the study by Cheng et al. [7], the authors presented insufficient information regarding the first domain, concerning the randomization process. The case-control study [24] presented critical and serious bias regarding confounding domains and patient selection, respectively. All of the other domains in all the included studies were well described.

## 4. Discussion

The aim of the present systematic review was to determine the effectiveness of a caries management by risk assessment tool (CAMBRA protocol) in decreasing the incidence of dental caries lesions and bacterial load.

The successful introduction of a caries management by risk assessment tool in daily clinical practice requires not only patient information and education, the identification of risk and protective factors, and the use of effective diagnostic methods, but also scientific evidence that ensures the most successful outcome for the patient. However, although the CAMBRA protocol offers a minimal intervention strategy, the identification of risk and protective factors, and an individualized treatment plan for the patient, the scientific evidence associated with this tool is limited.

Of the three studies that met the previously established inclusion criteria, only Cheng et al. [7] reported results with statistically significant differences. However, these results are related to intergroup (control/intervention) and not intragroup (baseline/follow-up) comparisons, which may constitute an important bias. In the study by Cheng et al. [7], the authors also failed to provide a complete description of the randomization process, namely regarding the randomization of the allocation sequence. It also presented bias in the effect of assignment to intervention since participants, clinicians, and people delivering the interventions were aware of the participants’ assigned intervention. These are important biases that ought to be considered when evaluating the methodological quality of the studies since the lack of blind participants and physicians may lead to an overestimation of the results.

In addition, one of the included studies [21] was a retrospective observational study that presented critical and serious bias regarding confounding domains and patient selection, respectively. These results in the bias assessment risk were somehow expected since one of the main disadvantages of this type of study design is the limited control the authors have over data collection since the information may be inaccurate or inconsistently evaluated among participants [25]. These biases create a reduction in the internal validity of the studies and, as such, should be considered.

The use of a placebo becomes essential for an accurate assessment of the effect of the CAMBRA protocol. The fact that changes are made to the daily oral hygiene routine only for patients of the intervention group may make these patients more aware, and it can positively affect the outcome, and the apparent effect size of the active intervention may be overestimated (Hawthorne effect) [26]. Rechmann et al. [23] were the only authors to use placebos in the control group, and they did not report statistically significant differences between groups regarding the incidence of dental caries lesions and battery load.

The identification of risk and preventive factors for dental caries as well as the information and education of the patients are critical aspects that contribute to their motivation and that lead them to adopt positive changes in their oral hygiene routine, which can dictate a change in the incidence of caries lesions and salivary bacterial load. As such, an intervention group, with no application of the CAMBRA protocol but, in turn, that focused on patient information and education, should be included in studies aiming at evaluating the effectiveness of this type of tool [27].

Regarding protocols, Rechmann et al. [23] were the only authors to report the protocol used in each group (control and intervention—low, medium, and high risk). In addition, Cheng et al. [7] and Kriegler et al. [24] did not mention the use of xylitol candies despite the fact that the original CAMBRA protocol indicated these for patients at moderate, high, and extreme risk for dental caries. Although the authors did not use the 2019/2021 CAMBRA protocol [16,17], in this update, xylitol is no longer listed as a protective factor as the evidence for its use is limited.

The fact that no results were reported for the 2019/2021 CAMBRA protocol [16,17] is justified by the fact that these are recent updates, a fact which has not yet allowed studies with adequate follow-up periods to be completed.

There is a clear need for further studies with adequate follow-ups, placebo controls, and patient education groups that evaluate not only the complete CAMBRA protocol, but also the effect of each of its components individually. This individual assessment will allow a better understanding of the influence that each component may have on the outcome, as well as the validation of their joint use. In addition, the protocol for children under age 6 was not evaluated by any of the included studies, which is a huge gap in its validation.

## 5. Conclusions

There is currently no scientific evidence to recommend the integration of the CAMBRA protocol into clinical practice. The results reported by the studies included in the systematic review do not suggest a decrease in the incidence of dental caries lesions and/or bacterial load. There is a clear need for the development of new clinical studies, with an adequate methodology and follow-up, both for the CAMBRA protocol and for its components individually.

## Figures and Tables

**Table 1:** PICO (Population, Intervention, Control, Outcome) strategy.

Parameter	Description
Population (P)	Adults and children
Intervention (I)	Dental caries treatment + CAMBRA protocol
Control (C)	Dental caries treatment
Outcome (O)	Dental caries incidence and/or oral bacterial load

**Table 2:** Search keys used in the different databases.

Database	Search Keys
PubMed	(CAMBRA) OR (“Caries Management by Risk Assessment”)
Cochrane Library	#1 CAMBRA #2 “Caries Management by Risk Assessment” #3 #1 OR #2
Web of Science	CAMBRA (All Fields) OR “Caries Management by Risk Assessment” (All Fields)
Scopus	(TITLE-ABS-KEY (CAMBRA) OR TITLE-ABS-KEY (“Caries Management by Risk Assessment”)
Embase	(CAMBRA) OR (“Caries Management by Risk Assessment”)

**Table 3:** Inclusion and exclusion criteria.

Criteria	Description
Inclusion Criteria	Clinical studies evaluating the incidence of dental caries lesions and/or reduction of cariogenic bacterial load (*Streptococcus mutans* and/or *Lactobacilli* spp.)
	Intervention group that follows the CAMBRA protocol
	Existence of a control group
Exclusion Criteria	Reviews, animal and cellular studies, letters, clinical cases, comments, and abstracts

**Table 4:** Results of the articles included in the systematic review.

Author/Year	Type of Study	Groups (*n*)	Products (Based on Individual Risk Assessment)	Age (Mean)	Follow-Up (Months)	Dental Caries	Bacterial Load (CFU/mL, Log_10_)
Cheng et al., 2015 [7]	Randomized clinical trial	G_1_: Control (52) G_2_: CAMBRA (57)	G_2_ Chlorhexidine mouthwash (0.12%); Sodium fluoride toothpaste (1.100 ppm); Sodium fluoride mouthwash (0.05%); Sodium fluoride gel (1.1%)	40	24	DMFS increment = 0 G_1_ = 7.7% G_2_ = 12.3% (*p* = 0.32) DMFS increment G_1_ = 4.6 ± 4.1 G_2_ = 3.5 ± 3.5 (*p* = 0.02)	*Streptococcus mutans* Baseline: G_1_ = 4.51 ± 1.29; G_2_ = 4.27 ± 1.42 (*p* = 0.37) 12 months: G1 = 4.58 ± 1.49; G_2_ = 3.26 ± 1.95 (*p* < 0.01) *Lactobacillus* spp. Baseline: G_1_ = 3.71 ± 1.98; G_2_ = 3.60 ± 1.95 (*p* = 0.76) 12 months: G_1_ = 3.28 ± 2.00; G_2_ = 2.92 ± 2.10 (*p* = 0.25)
Rechmann et al., 2018 [23]	Randomized clinical trial	G_1_: Control (239) G_2_: CAMBRA (221)	G_1_ Nonfluoridated mouthwash; Fluoridated toothpaste (1.100 ppm); Placebo varnish; Sorbitol candies G_2_ Chlorhexidine mouthwash (0.12%); Fluoridated toothpaste (1.100 ppm); Fluoridated toothpaste (5.000 ppm); Fluoride mouthwash (0.05%); Fluoridated varnish; Xylitol candies	36	24	Cavities on radiograph into dentin Baseline: G_1_ = 17.1%; G_2_ = 17.5% (*p* = 0.33) 6 months: G_1_ = 7.0%; G_2_ = 6.3% (*p* = 0.86) 12 months: G_1_ = 4.3%; G_2_ = 10.3% (*p* = 0.33) 18 months: G_1_ = 7.5%; G_2_ = 3.7% (*p* = 0.49) 24 months: G_1_ = 15.4%; G_2_ = 0% Proximal enamel lesions on radiograph Baseline: G_1_ = 24.8%; G_2_ = 27.7% (*p* = 0.45) 6 months: G_1_ = 21.1%; G_2_ = 16.5% (*p* = 0.42) 12 months: G_1_ = 17.0%; G_2_ = 17.6% (*p* = 0.79) 18 months: G_1_ = 22.5%; G_2_ = 18.5% (*p* = 0.35) 24 months: G_1_ = 17.9%; G_2_ = 16.3% (*p* = 0.90) Active white spot lesions Baseline: G_1_ = 18.1%; G_2_ = 19.7% (*p* = 0.48) 6 months: G_1_ = 12.3%; G_2_ = 10.1% (*p* = 0.71); 12 months: G_1_ = 19.1%; G_2_ = 11.8% (*p* = 0.26) 18 months: G_1_ = 0.10%; G_2_ = 7.4% (*p* = 0.93) 24 months: G_1_ = 12.8%; G_2_ = 11.6% (*p* = 0.75)	_
Kriegler et al., 2021 [24]	Case-control	G_1_: Control (100) G_2_: CAMBRA (107)	G_2_ Chlorhexidine mouthwash (0.12%); Fluoridated toothpaste (5.000 ppm); Sodium fluoride mouthwash (0.05%)	69	12	Dental caries incidence Baseline: G_1_ = 49.0%; G_2_ = 41.1% (*p* = 0.27) 12 months: G_1_ = 41.1%; G_2_ = 18.7% (*p* = 0.10)	-

G: group.

## Data Availability

Not applicable.
